# Staged Hybrid Techniques With Straightforward Bypass Surgery Followed by Flow Diverter Deployment for Complex Recurrent Middle Cerebral Artery Aneurysms

**DOI:** 10.3389/fsurg.2022.824236

**Published:** 2022-02-02

**Authors:** Jun Tanabe, Ichiro Nakahara, Shoji Matsumoto, Jun Morioka, Akiko Hasebe, Sadayoshi Watanabe, Kenichiro Suyama, Kiyonori Kuwahara

**Affiliations:** Department of Comprehensive Strokology, Fujita Health University School of Medicine, Toyoake, Japan

**Keywords:** aneurysm, bypass, endovascular therapy (EVT), hybrid technique, middle cerebral artery aneurysms

## Abstract

**Background:**

Recurrent complex middle cerebral artery (MCA) aneurysms after combined clipping and endovascular surgery are challenging, and if conventional techniques are adapted, advanced surgical, endovascular, and a combination of both techniques are often required. For such complex aneurysms, safe and effective straightforward techniques for all neurovascular surgeons are warranted. We describe the details of staged hybrid techniques with straightforward bypass surgery followed by flow diverter deployment in a patient with complex MCA aneurysm.

**Illustrative Case:**

A 69-year-old woman presented with left recurrent large MCA aneurysm enlargement 25 years after direct surgery and coil embolization for ruptured aneurysm. The recurrent MCA aneurysm had large and complex morphology and was adhering to the brain tissues. Therefore, it was unsuitable to treat such aneurysm with conventional surgical and endovascular techniques with a high risk of morbidity. We performed (1) M2 ligation following superficial temporal artery-M2 bypass and (2) flow diverter deployment assisted with coil packing in two sessions. Three months after the second session, the aneurysm was completely occluded with endothelialization of the neck. Angiographic findings revealed no recurrence 12 months after the treatment.

**Conclusions:**

Staged hybrid techniques with straightforward bypass surgery followed by flow diverter deployment may be a safe and effective treatment for complex recurrent MCA aneurysms.

## Introduction

Recurrent complex middle cerebral artery (MCA) aneurysms after clipping and endovascular surgery are challenging because advanced direct surgery and endovascular techniques are often required (1–8). For such complex aneurysms, safe and effective straightforward techniques for all neurovascular surgeons are warranted. We describe a staged hybrid technique characterized by M2 ligation following superficial temporal artery (STA)-M2 bypass and flow diverter (FD) deployment assisted with coil packing for recurrent complex large MCA aneurysm.

## Illustrative Case

### Clinical Presentation

**Figure 1:**
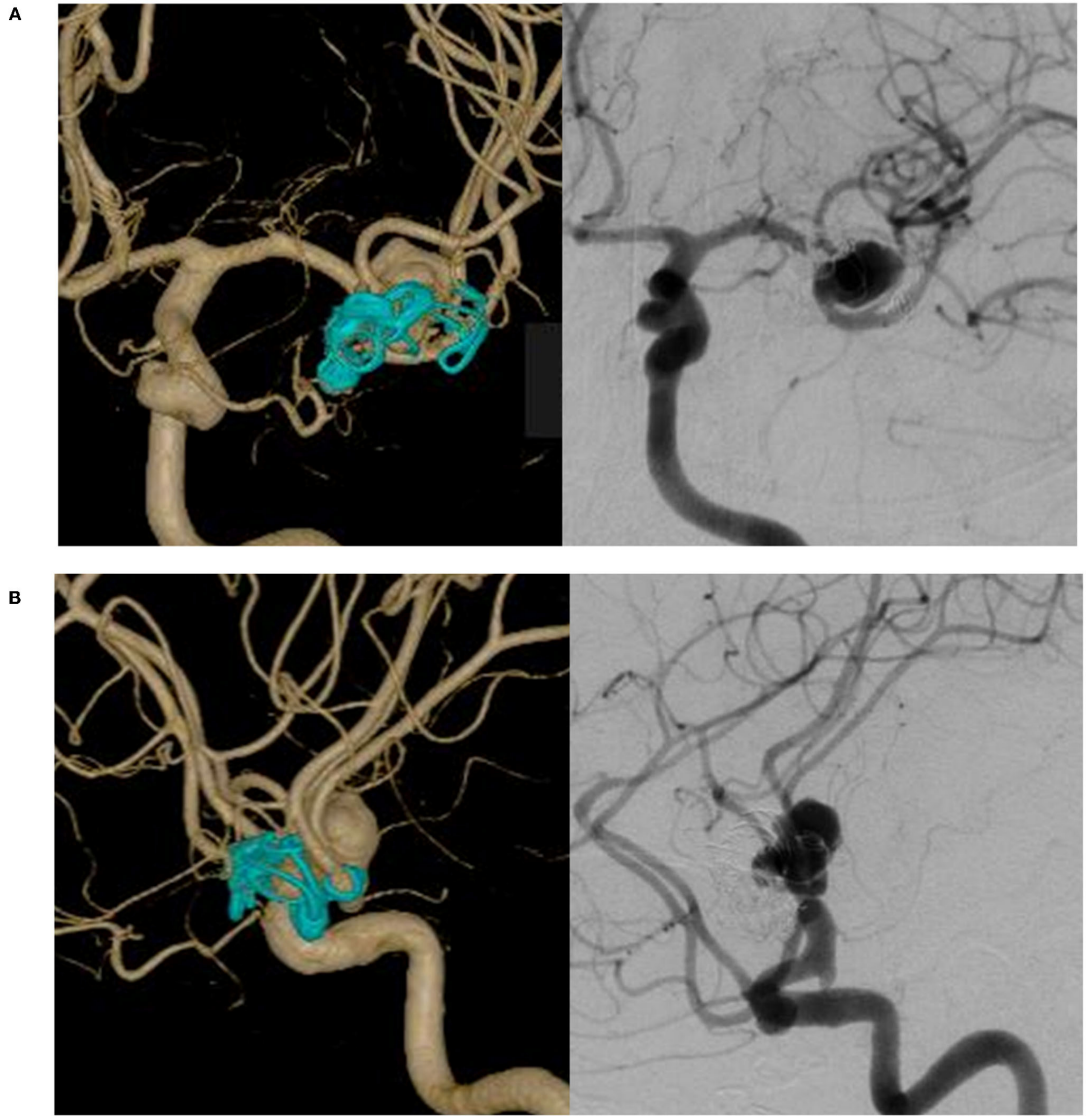
Left internal carotid angiography reveals recurrent large middle cerebral artery aneurysm unrelated to a previous coil. **(A)** In an antero-posterior view. **(B)** In a lateral view.

A 69-year-old woman presented with left recurrent large MCA aneurysm enlargement 25 years after direct surgery and coil embolization for a ruptured aneurysm. For the initial treatment of a ruptured aneurysm 25 years previously, neck clipping was attempted, but she received only a coating due to the aneurysm's unclippable morphology. Subsequently, coil embolization was performed using a simple technique. Nine years after the initial treatment, an additional coil was inserted, owing to coil compaction. Twenty-three years after the initial treatment, regrowth of the aneurysm was detected; however, conservative treatment was selected because of treatment difficulty. Two years later, she was referred to us for treatment of further rapid enlargement. Angiography revealed aneurysm regrowth (12 × 8 × 6 mm) unrelated to the previous coil mesh (Figure 1). The STA-parietal branch was sacrificed at initial treatment before 25 years, and the STA-frontal branch (FB-STA) was preserved with a small caliber. It was unsuitable to treat such an aneurysm with conventional surgical or endovascular techniques because previous coil mesh protruded from outside of the aneurysm, and firm adhesion of the aneurysm to the surrounding brain tissue was a concern. Hybrid-combined therapy with direct surgery and endovascular treatment was performed in two sessions: (1) FB-STA and M2-superior trunk, originating from the aneurysm with a steep angle, followed by ligation of the M2-superior trunk proximal to the bypass, and (2) FD deployment following coil embolization.

### Treatment and Technique

**Figure 2:**
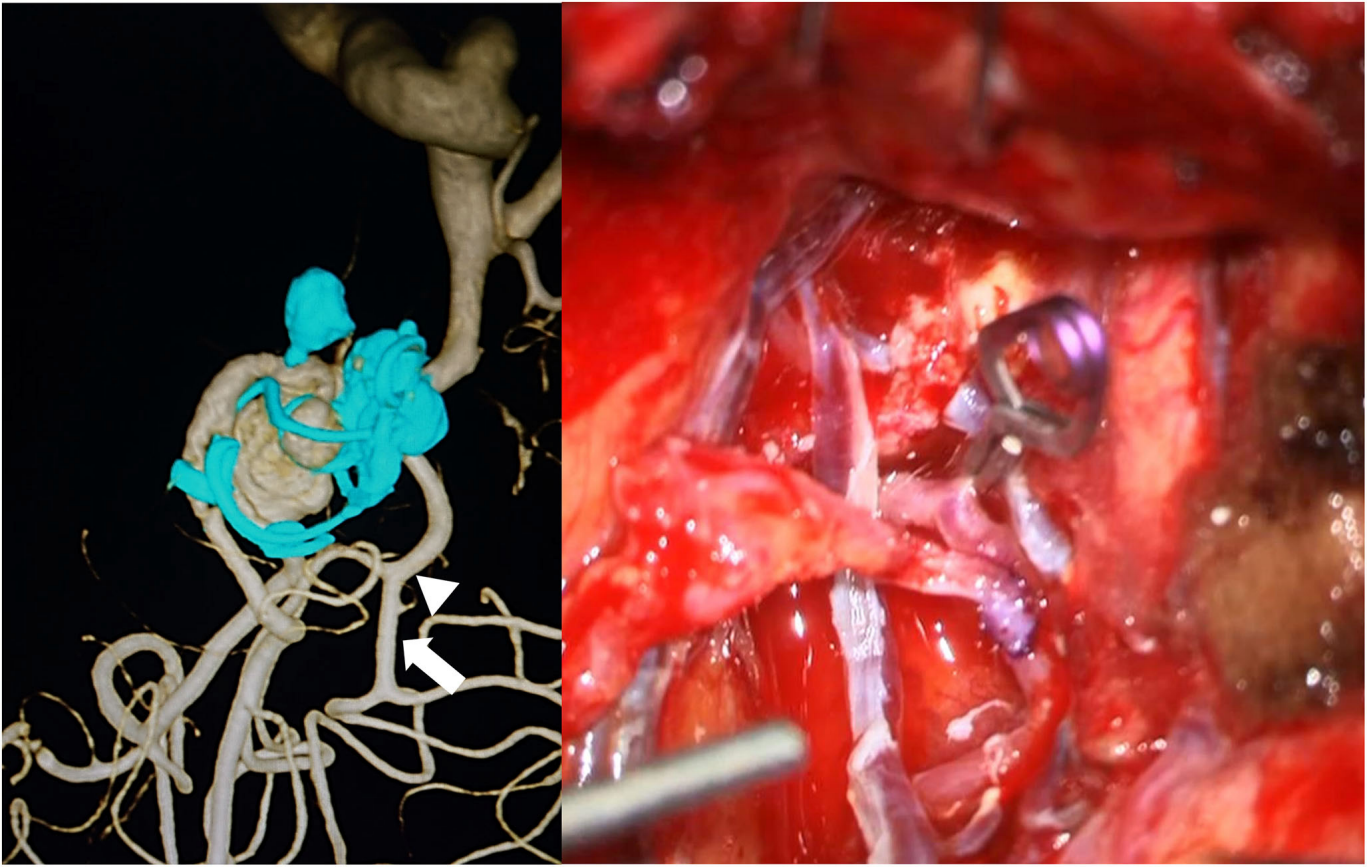
Left: Preoperative three-dimensional rotational angiography, describing the aneurysm estimated for an operative view. The arrow indicates the bypass site, and the arrowhead indicates the ligation point. Right: A frontal branch of the superficial temporal artery and M2 bypass followed by ligation of M2 proximal to bypass.

**Figure 3:**
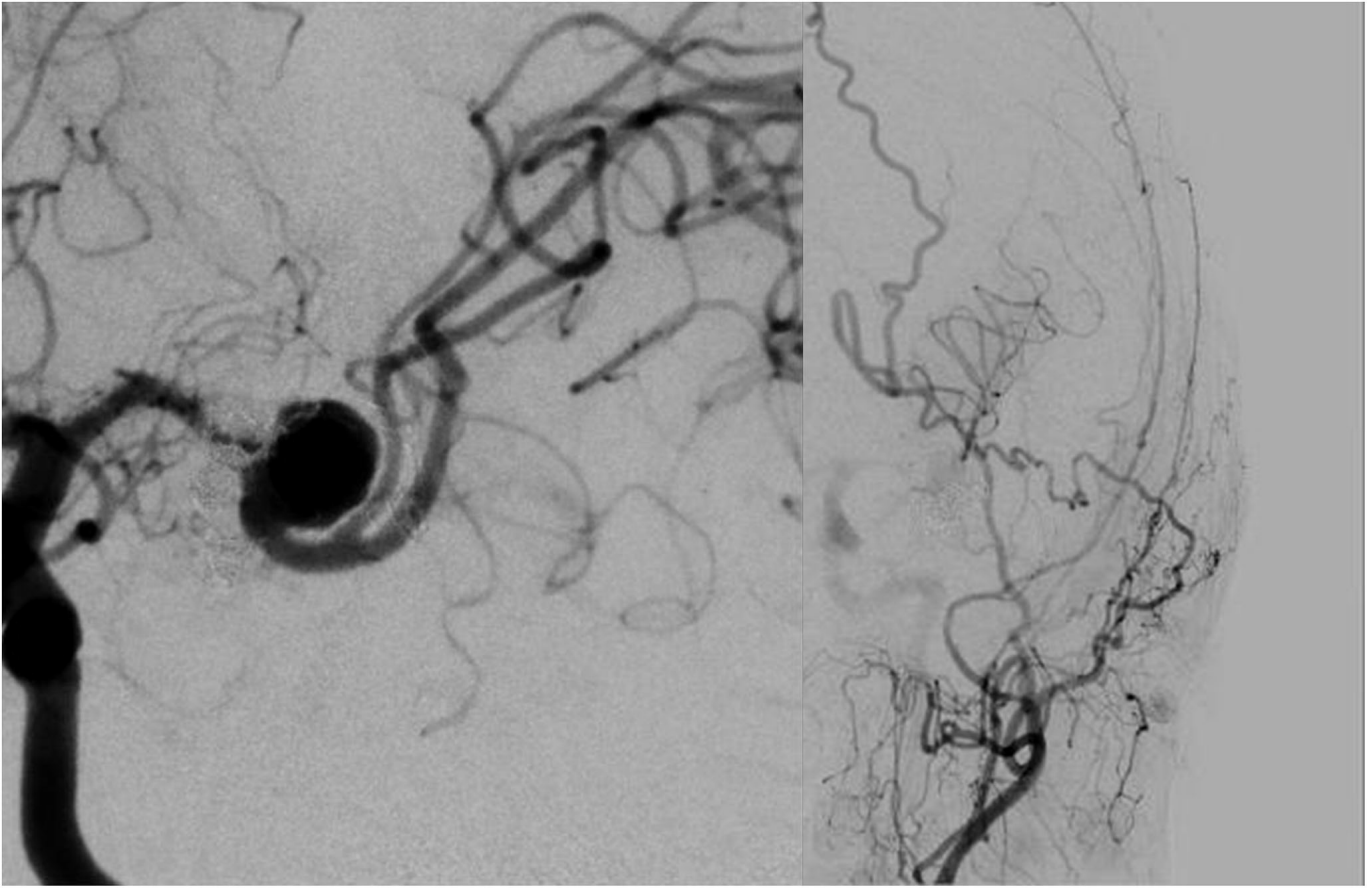
Postoperative angiography of first session reveals sacrifice of superior trunk “that is transformation to side-wall-type aneurysm” (left) and good patency of bypass (right).

In the first session, frontotemporal craniotomy was performed using a previous skin incision and bone flap. FB-STA was harvested with a direct cut down on the artery behind the skin flap. The Sylvian fissure was widely dissected, revealing the M2/M3 portion (9, 10). It was difficult to fully expose the aneurysm because the aneurysm was strongly adherent to the surrounding brain tissue, coating material, and previously extruded coil mesh. Therefore, the superior branch of the M2 was exposed. FB-STA and M2 bypass were performed, and the patency of the bypass was confirmed using indocyanine green videoangiography. Thereafter, the M2 proximal to the bypass was ligated (Figure 2). Trans-cranial motor-evoked potential (MEP) was intact for 10 min after M2 ligation, and cranial closure was performed. The MEP was intact until the end of the surgery, approximately 60 min after M2 ligation. Thus, the recurrent MCA aneurysm was transformed to a side-wall-type aneurysm (Figure 3).

**Figure 4:**
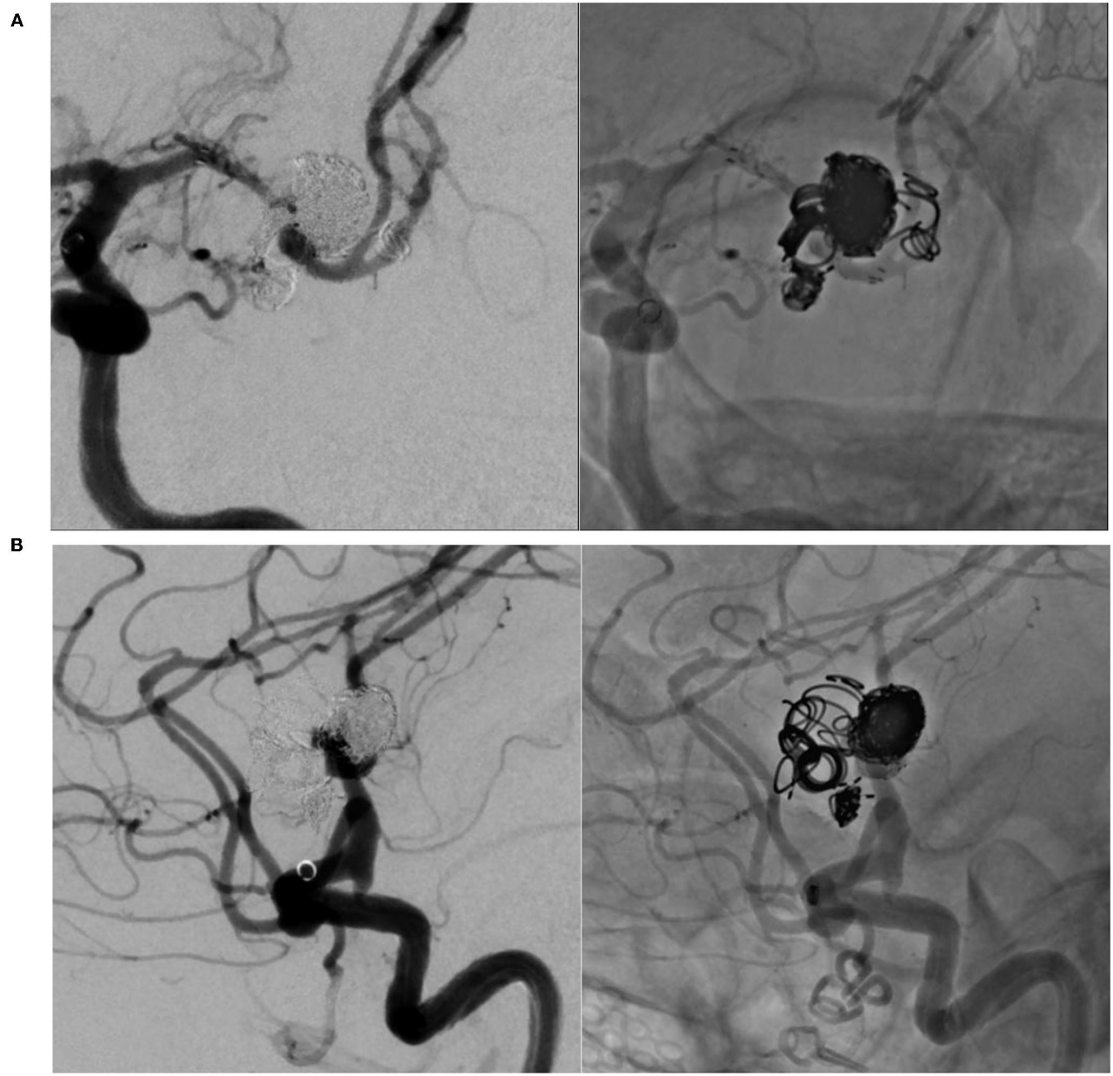
**(A)** Flow diverter deployment following coil insertion in the antero-posterior view. Coil embolization is performed with ten detachable coils (Barricade complex framing; Blockade Medical, Irvine, California, USA, Axium Prime 3D; Medtronic). **(B)** In a lateral view. The aneurysm underwent a subtotal filling, and the artery, where flow diverter (FD) was deployed, was slightly straightened.

In the second session, 3 weeks after the first session, endovascular therapy was performed. The patient received dual antiplatelet therapy for 2 weeks before the procedure. An 8-Fr guiding catheter was introduced into the left internal carotid artery. A 6-Fr distal access catheter (SOFIA SELECT; MicroVention-Terumo, Tustin, California, USA) was navigated into the IC top. Coils were roughly inserted through the microcatheter (Phenom 17; Medtronic, Minneapolis, MN, USA) with balloon assistance (Scepter XC; 4. mm in diameter, 11 mm in length; Microvention-Terumo). The balloon catheter was exchanged with a microcatheter for FD deployment (Headway 27; MicroVention-Terumo) with a long microguide wire (CHIKAI, 300-cm length; Asahi Intecc, Nagoya, Aichi, Japan). An FD [flow redirection endoluminal device (FRED) 3.5 mm in diameter, 13/11 mm in length; MicroVention-Terumo] was deployed from the M2 posterior trunk to the M1, covering the aneurysm neck. Immediately after FD deployment, the aneurysm was subtotally filled (O' Kelly-Marotta grading scale B). The aneurysm became even more side wall type because FD deployment straightened the artery from the M2 posterior trunk to M1 (Figure 4). The operative technique is presented in the surgical video (Supplementary Material).

### Outcomes and Follow-Up

**Figure 5:**
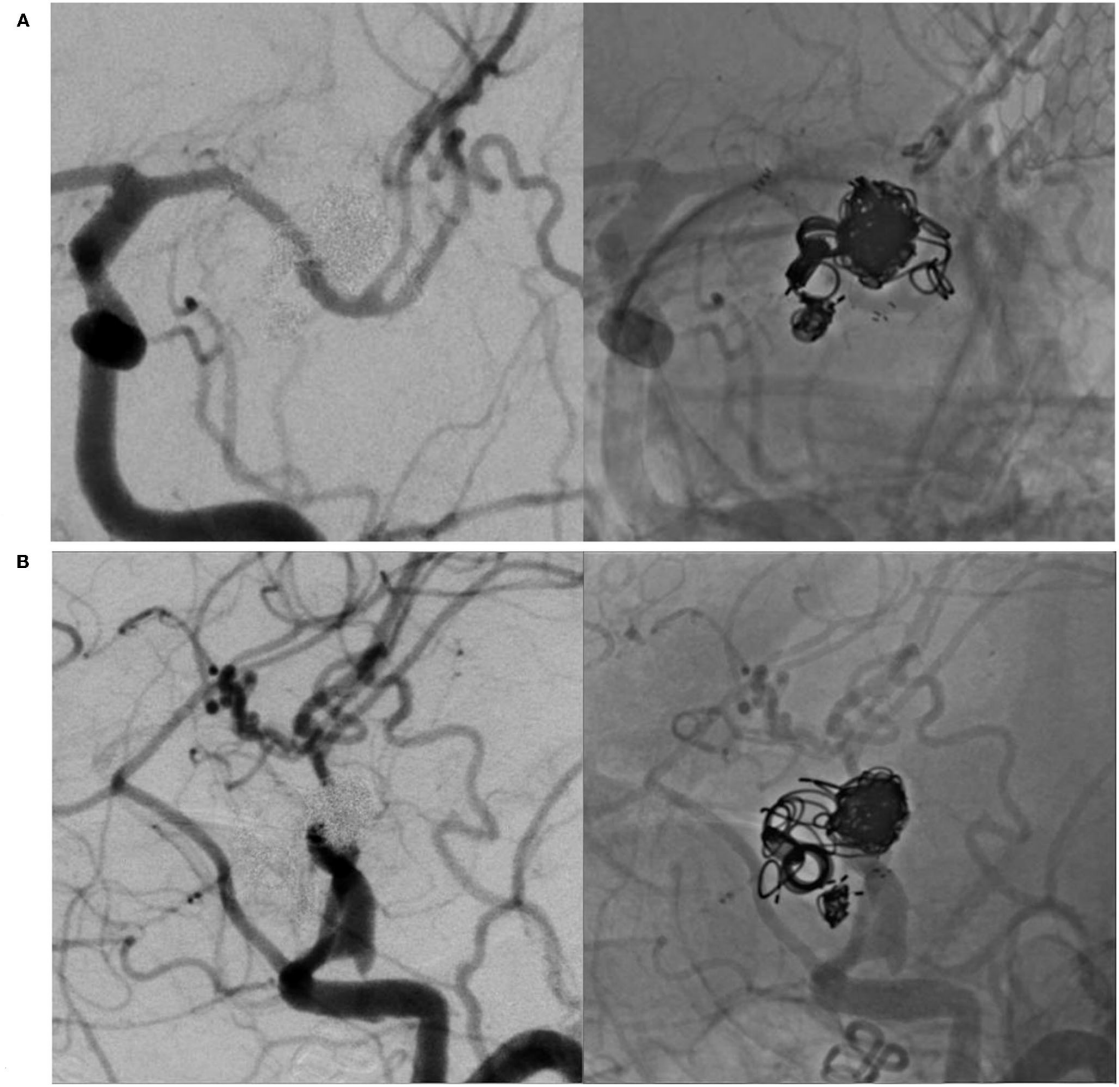
Left internal carotid angiography 3 months after treatment reveals complete occlusion of the aneurysm with endothelialization of the neck. **(A)** In an antero-posterior view. **(B)** In a lateral view.

The patient recovered without symptomatic complications in both the procedures. No high-intensity signal, including the territory of the perforator, such as the lenticulostriate artery (LSA), appeared on the diffusion-weighted image. Three months after the second treatment session, the aneurysm was completely occluded with neck endothelialization (O'Kelly-Marotta grading scale D) by follow-up digital subtraction angiography (Figure 5) (11). Angiography revealed no recurrence 12 months after the treatment. Dual antiplatelet therapy was maintained for 6 months, and single-agent therapy was discontinued 12 months after the treatment.

## Discussion

### Principle of a Present Staged Strategy

**Figure 6:**
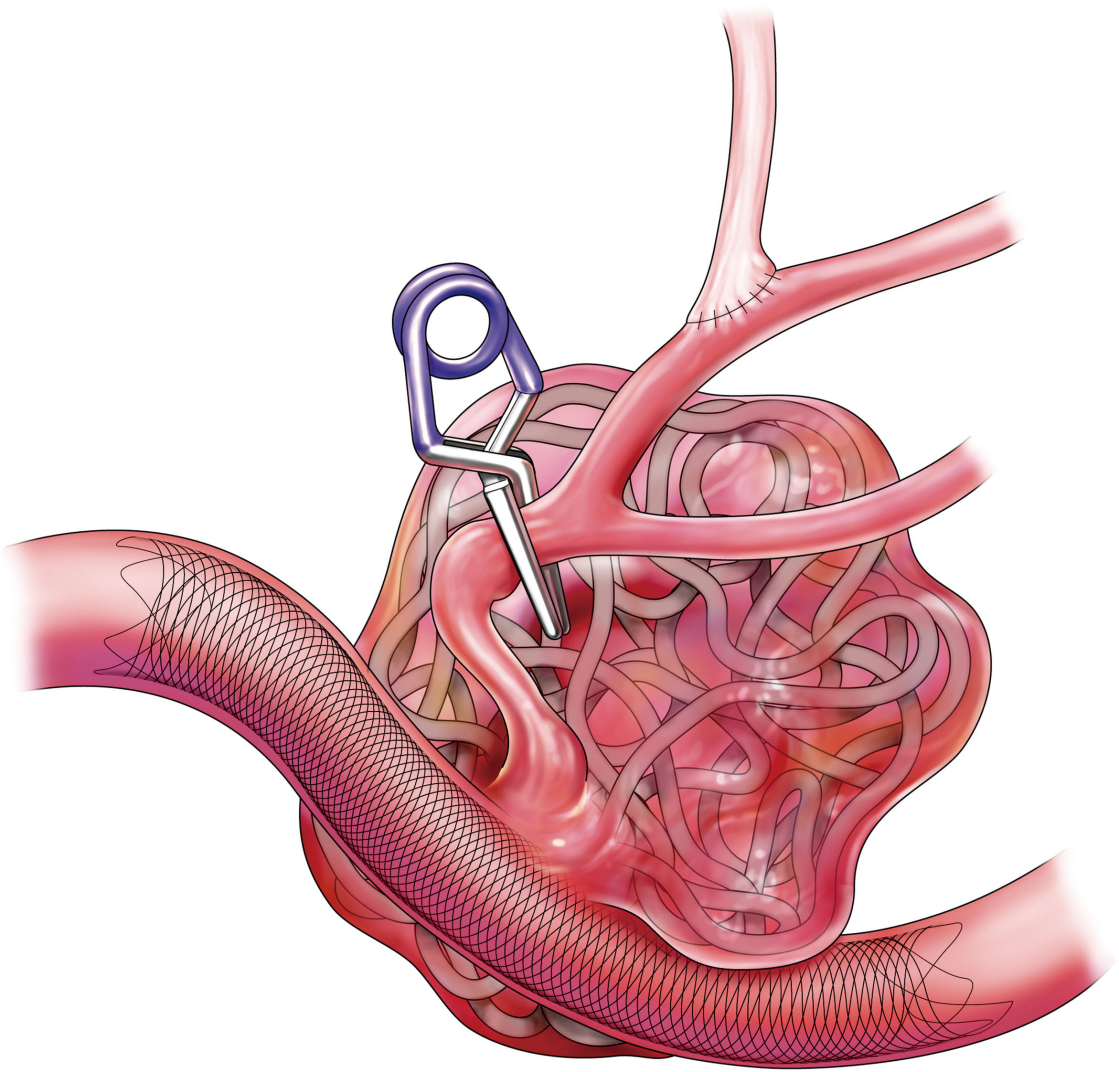
Schematic illustration depicting the present techniques.

The present study described staged hybrid techniques characterized by debranching the artery planned to be jailed by FD using low-flow bypass and FD deployment for complex bifurcation aneurysms (Figure 6). To promote early healing, adjunctive coil embolization was performed because the presented aneurysm had short-term aggressive enlargement. Thus, the aneurysm was completely occluded in the short term.

Combined surgical and endovascular techniques for complex aneurysms have been reported (12, 13). Before FD treatment, transformation from a bifurcation aneurysm to a side wall aneurysm by side-to-side M2 anastomosis followed by coil embolization of the M2 has been a minimally invasive approach (14). Technical characteristics, safety, and effectiveness have been reported regarding side-to-side anastomosis (15, 16). However, it is difficult to make a suitable surgical field for side-to-side anastomosis in retreatment situations due to tissue adhesion. Side-to-side anastomosis requires advanced technical skills and risks the occlusion of both arteries, resulting in major ischemic complications. In contrast, low-flow bypass sufficiently supplies blood flow to one side of the M2 territory with electrophysiological monitoring and is a familiar technique for many neurovascular surgeons. In addition, technical advancements in endovascular devices have allowed easier and safer FD deployment. Therefore, among the various combined surgical and endovascular techniques for complex recurrent MCA aneurysms, this staged hybrid technique with low-flow bypass and FD deployment may comprise the preferred treatment option.

### Concerns of Conventional and Another Recent Advanced Treatment

Microsurgical clipping and endovascular treatment are challenging in complex aneurysms, including aneurysms that lack a neck, which have a large size, and have been treated previously. Consequently, in the conventional strategy, trapping or proximal occlusion for flow alteration was adopted following bypass surgery, including high flow bypass using an interposition graft such as a radial artery or saphenous vein. However, uncertain aneurysm occlusion in flow alteration treatment, cerebral infarction due to graft occlusion, and hyperperfusion for high-flow bypass have been a concern (1–3). In contrast, endovascular techniques, including even FD deployment, have the risk of incomplete occlusion and recurrence, especially in complex large MCA bifurcation aneurysms (4–7, 17).

In recent years, novel endovascular devices have become available for wide-neck bifurcation aneurysms, such as PulseRider and Woven EndoBridge devices (18, 19). However, the use of these devices for aneurysms with a steep parent artery-aneurysm angle, previous coil mesh, or thrombus is contraindicated.

### Advantage of FD Deployment Following Transformed Aneurysm Morphology

We proposed that FD should be deployed following the sacrifice of the jailed M2 after STA-MCA bypass for complex MCA aneurysm. FD has been increasingly used in endovascular treatment of distal anterior circulation aneurysms for treatment efficacy (5, 7, 20, 21). Especially for giant and large aneurysms, FD treatment provides a higher complete occlusion rate at follow-up and a lower recurrence rate than stent-assisted coiling, while the immediate occlusion rate is similar for both treatments (residual aneurysm in almost cases) (22). In the FD treatment for MCA aneurysms, subsequent infarction of the LSA territory after FD deployment is not a major concern under suitable antiplatelet management based on previous reports and clinical experiences of FD in more than a decade (23). However, MCA location has been an independent factor associated with lower occlusion rate, and the FD treatment for MCA bifurcation aneurysms has not been suitable unless conventional treatment options are ineffective (4–6). In an experimental study, incomplete occlusion following FD deployment for bifurcation aneurysms can be caused by persistent flow of the jailed artery (17). Therefore, combined treatment with branch occlusion for FD deployment is recommended. Transforming a bifurcation aneurysm to a sidewall type using our staged strategy facilitates neo-endothelialization after FD deployment, resulting in long-term stability of aneurysm occlusion.

## Conclusion

We present a case in which staged hybrid techniques with straightforward bypass surgery followed by FD deployment were introduced to successfully obliterate complex recurrent MCA aneurysms 25 years after direct surgery and coil embolization. This strategy may be safe and effective, and, comprising the preferred treatment option for complex MCA aneurysms, it could be widely adopted by neurovascular surgeons.

## Data Availability Statement

The raw data supporting the conclusions of this article will be made available by the authors, without undue reservation.

## Ethics Statement

Ethical review and approval was not required for the study on human participants in accordance with the local legislation and institutional requirements. The patients/participants provided their written informed consent to participate in this study. Written informed consent was obtained from the individual(s) for the publication of any potentially identifiable images or data included in this article.

## Author Contributions

JT and IN contributed conception and design of the study and wrote sections of the manuscript. JT, IN, SM, JM, AH, SW, KS, and KK contributed to the acquisition and analysis of the data. JT wrote the first draft of the manuscript. All authors contributed to manuscript revision, read, and approved the submitted version.

## Conflict of Interest

IN reports grants from Kaneka Medix, Medtronic, and Nipro, consultancy from Kaneka Medix, and Medicos Hirata, outside the submitted work. The remaining authors declare that the research was conducted in the absence of any commercial or financial relationships that could be construed as a potential conflict of interest.

## Publisher's Note

All claims expressed in this article are solely those of the authors and do not necessarily represent those of their affiliated organizations, or those of the publisher, the editors and the reviewers. Any product that may be evaluated in this article, or claim that may be made by its manufacturer, is not guaranteed or endorsed by the publisher.

## Supplementary Material

Supplementary Video Staged hybrid techniques with straightforward bypass surgery followed by flow diverter deployment for complex recurrent middle cerebral artery aneurysms: The operative technique demonstration of the illustrative case.
